# Mechanism underlying the inhibitory effect of Apelin-13 on glucose deprivation-induced autophagy in rat cardiomyocytes

**DOI:** 10.3892/etm.2013.902

**Published:** 2013-01-17

**Authors:** HUI JIAO, ZHI ZHANG, QINGHUA MA, WEI FU, ZIDONG LIU

**Affiliations:** Department of Cardiology, The Third Affilated Hospital of Liaoning Medical University, Jinzhou 121001, P.R. China

**Keywords:** Apelin-13, autophagy, cardiomyocytes, glucose deprivation, light chain 3, PI3K/Akt/mTOR pathway

## Abstract

The aim of the present study was to investigate the effect of Apelin-13 on cardiomyocyte autophagy and to determine the underlying mechanism of this effect. To establish an autophagic model system, the cardiomyocytes of Sprague Dawley rats (postnatal day 3) were cultured and divided into five groups: normal control (Co), glucose deprivation (GD), GD+Apelin-13, GD+Apelin-13 treated with the Akt-specific inhibitor triciribine (GD+Apelin-13+Triciribine) and triciribine alone (Triciribine). The intracellular autophagosomes were then observed using transmission electron microscopy (TEM) and the expression levels of cellular autophagy-related protein microtubule-associated protein 1 light chain 3 (LC3), phosphatidylinositol-3-kinase (PI3K) and mammalian target of rapamycin (mTOR) protein were measured using western blotting. Compared with the Co group, the ratio of LC3-II/LC3-I increased significantly in all treatment groups, with the exception of the Triciribine group (P<0.05). Compared with the GD group, the ratio of LC3-II/LC3-I was significantly decreased, and the PI3K and mTOR expression was significantly enhanced in the GD+Apelin-13 and GD+Apelin-13+Triciribine groups (P<0.05). Compared with the GD+Apelin-13 group, the ratio of LC3-II/LC3-I increased significantly (P<0.05) and the PI3K expression remained unchanged in the GD+Apelin-13+Triciribine group (P>0.05), but mTOR expression was significantly reduced (P<0.05). GD led to increased numbers of autophagosomes and augmented the LC3-II/LC3-I ratio (P<0.05). Apelin-13 pretreatment attenuated GD-induced cardiomyocte injury, decreased the autophagosome number and the ratio of LC3-II/LC3-I (P<0.05), enhanced PI3K activity (P<0.05) and upregulated the phosphorylation levels of the Akt and mTOR proteins (P<0.05). The Akt-specific inhibitor triciribine weakened the protective role of Apelin-13 (P<0.05). To a certain extent, Apelin-13 inhibited GD-induced cardiomyocyte autophagy, which may be related in part to the activation of the PI3K/Akt/mTOR signaling pathway.

## Introduction

Autophagy is a prevalent phenomenon in eukaryotic cells and occurs following myocardial ischemia and subsequent reperfusion (1). Low levels of autophagy play a protective role in cardiomyoctyes, whereas high levels or chronic autophagy are able to facilitate cell injury and result in the death of cells that cannot be repaired (2). Determining how to effectively inhibit autophagy and attenuate myocardial damage has become a cardiovascular disease research hotspot (3,4). Apelin is highly expressed in the cardiovascular system, where it dilates blood vessels to reduce blood pressure, improves heart function, inhibits cardiomyocyte apoptosis and protects against myocardial ischemia reperfusion injury (5,6). We have previously shown that Apelin-13 also inhibits cardiomyocyte autophagy (7), but the underlying mechanism remains unclear. In the present study, the possible mechanism responsible for autophagy following glucose deprivation (GD) in cardiomyoctyes was investigated.

Autophagy is important for clearing injured organelles following hypoxia-ischemia, nutritional deficiencies, oxidative stress and infection. While autophagy plays a protective role, excessive autophagy may result in type II programmed cell death (2). Hence, chronic autophagy may lead to irreversible damage. The pathways involved in autophagy progression are complicated, with three pathways positively identified thus far: i) type I phosphatidylinositol-3-kinase (PI3K)/Akt signaling, ii) mammalian target of rapamycin (mTOR) signaling and iii) type III PI3K signaling. The first pathway inhibits autophagy. mTOR signaling plays a significant regulatory role in cell growth and receives multiple upstream signals, including those of type I PI3K, IGF-1/2 and mitogen-activated protein kinase (MAPK). Moreover, it inhibits autophagy by perceiving changes in nutrient and energy levels. The structure of type III PI3K is similar to that of type I PI3K, however, it presents with a reversed role. Autophagosome formation may be interfered with or blocked using PI3K inhibitors, including 3-MA, wortmannin and LY294002 (8).

Tatemoto *et al*(9) first extracted and purified Apelin from calf stomach secretions using reverse pharmacology. Apelin was determined to be an endogenous ligand of the orphan G protein-coupled receptor angiotensin type 1 receptor-associated protein (APJ). Apelin is expressed ubiquitously, with particularly high levels in the cardiovascular system and with the ability to dilate blood vessels to reduce blood pressure, improve heart function, inhibit cardiomyocyte apoptosis and protect against myocardial ischemia reperfusion injury (3,5,6). We have previously shown that Apelin-13 also inhibits cardiomyocyte autophagy (7), however the underlying mechanism remains unclear. Another study suggested that PI3K/Akt signaling may be involved (10). In the present study, the effect of Apelin-13 on autophagy was studied further and the role of PI3K/Akt signaling was assessed. Our previously established *in vitro* neonatal rat cardiomyocyte model of GD was employed (10) and transmission electron microscopy (TEM), PI3K activity assays and western blotting were performed. Collectively, our findings support the hypothesis that Apelin-13 inhibits cardiomyocyte autophagy by modulating PI3K/Akt signaling.

## Materials and methods

### Reagents

Specimens were obtained from Sprague Dawley (SD) 1–3 day postnatal rat pups (Experimental Animal Center of Liaoning Medical College, Jinzhou, China). Dulbecco’s modified Eagle’s medium (DMEM):F12 (1:1) culture medium, calf serum, 5-bromodeoxyuridine (BrdU) and phosphate-buffered saline (PBS) balanced salt solution were all purchased from Hyclone (Logan, UT, USA). Trypsin (Beyotime Co., Jiangsu, China), Apelin-13 and triciribine (Sigma, St. Louis, MO, USA) were also used.

Anti-phosphotyrosine antibody was purchased from Santa Cruz Biotechnology (Santa Cruz, CA, USA.) Rabbit anti-human-β-actin, LC3, Akt, p-Akt (Ser473), mTOR and p-mTOR (Ser2448) antibodies were purchased from Cell Signaling Technology (Danvers, MA, USA). The study was approved by the ethical committee of The Third Affilated Hospital of Liaoning Medical University (Jinzhou, China).

### Culture and identification of myocardiocytes

The protocol to culture and identify the myocardiocytes was employed as described previous (11). The experiments were approved by the Institutional Animal Care and Use Committee. Briefly, SD rats at postnatal days 1–3 were sacrificed and their hearts removed under sterile conditions, rinsed in PBS balanced salt solution three times and then cut into 1-mm^3^ sections. The samples were placed into a magnetic stirrer at 37°C with 0.06% trypsin until they were completely digested. The digestion was stopped using calf serum. The resulting cell suspension was centrifuged at 1,200 rpm for 10 min and resuspended in DMEM:F12 (1:1) medium containing 15% fetal calf serum. The resulting single-cell suspension was filtered through a 200 mesh filter and the cell density was adjusted to 5×10^5^/ml prior to inoculation in a 25-cm^3^ flask. The flasks were then placed in an incubator at 37°C with 5% CO_2_. The fibroblasts were removed by replacing the culture medium after 90 min when the myocardiocytes had adhered to the flask. The cell suspension was added with 5-BrdU to a final concentration of 0.1 ml/l and adjusted to 5×10^5^/ml. The culture medium was replenished every 24 h. Subsequent to being cultured for 72 h, the cells that grew in a good condition with regular contraction were used for the experiments. Monoclonal antibodies against α-sarcomeric actin were used to characterize the myocardiocytes.

### Experimental groups and model establishment

The cardiomyocytes cultured for 72 h were divided into 5 groups: i) Control group, the culturing of the cells continued without any treatment for 12 h subsequent to changing the medium; ii) GD group, the culturing of the cells continued for 12 h subsequent to the replacement of the medium with a glucose-free DMEM:F12 (1:1) medium; iii) GD+Apelin-13 group, the cells were pretreated with 1 *μ*mol/l Apelin-13 for 30 min prior to the change to a glucose-free medium; iv) GD+Triciribine+Apelin-13 group, the cells were pretreated with 1 *μ*mol/l triciribine for 30 min and 1 *μ*mol/l Apelin-13 for 30 min in succession prior to the change to a glucose-free medium; and v) Triciribine group, the cells were pretreated with 1 *μ*mol/l triciribine for 30 min followed by 12 h in a normal culture medium.

### Autophagosome observation by TEM

Next, the culture medium was apirated and the cells were gently washed three times with PBS buffer prior to harvesting the cells with cell scratches. The samples were placed in a tube and centrifuged at 1,800 rpm for 30 min and the supernatant was carefully absorbed. The cells were then fixed with 3% glutaraldehyde and 1% osmium tetroxide for 3 h. Following rinsing with PBS for 30 min, the samples were dehydrated with ethanol and isopropanol, embedded in epoxy resin and prepared under a dissecting microscope. An ultrathin sectioning machine (Leica EM UC6, Leica Microsystems, Mannheim, Germany) was used to prepare the 1-*μ*m sections, then the samples were double stained with uranyl acetate and lead citrate and the cardiomyocytes were observed using TEM (JEM-1200EX, JEOL Ltd., Tokyo, Japan). Images were captured and 10 randomly selected fields of vision from each group were used to quantify the area of the autophagosomes to the total cytoplasmic area.

### PI3K activity assay using immunoprecipitation lipid kinase analysis

The PI3K activity was measured as previously described (12). Briefly, the total proteins were extracted and incubated with anti-phosphotyrosine antibodies for 1 h. The proteins were subsequently incubated with a G-protein-coated agarose bead suspension for 4–12 h. The G-protein-coated agarose beads were washed 3 times with a buffer containing 100 mM Tris (pH 7.5) and 500 mM LiCl, followed by a single wash with buffer containing 10 mM Tris-HCl, 100 mM NaCl, 1 mM EDTA and then a further single wash in the PI3K analysis buffer. The washed antigen-antibody complex was pre-incubated with 50 *μ*l PI3K buffer and 20 *μ*l lipid substrate mixture. The enzyme reaction was carried out using 10 *μ*l γ^32^P-ATP mixture. After 10 min, the reaction was quenched by adding an equal volume (80 *μ*l) of stop solution. The mixture was extracted twice using phosphorylated chloroform-methanol (1:1). The resulting mixture was analyzed using thin-layer chromatography and autoradiography. Radioactivity was detected using a FJ-2115 automatic liquid scintillation counter. The mean radioactivity in the control group was set as 100% and the radioactivity levels were compared among the groups.

### Western blotting for the detection of LC3, Akt, p-Akt, mTOR, p-mTOR and PI3K

The cardiomyocytes were washed twice in cold PBS then gently mixed in a lysis buffer [25 mM Tris-HCl (pH 7.4), 150 mM NaCl, 2 mM EDTA, 1% Triton-X-100, 1% sodium deoxycholate, 0.1% sodium dodecyl sulfate and protease inhibitor cocktail] and incubated for 20 min on ice. The samples were centrifuged at 12,000 rpm for 20 min at 4°C and the supernatant was collected and subjected to a bicinchoninic acid assay for protein quantification. Samples consisting of 50 *μ*g total protein were separated using sodium dodecyl sulfate polyacrylamide gel electrophoresis and the proteins were transferred to membranes using a semi-dry method. The membranes were then blocked for 1 h with 5% bovine serum albumin in PBS with Tween-20 (BSA-PBST) at room temperature. The blots were then incubated with primary antibodies against LC3, PI3K, Akt, p-Akt, mTOR, p-mTOR or β-actin at 4°C overnight. The next day, the blots were washed and incubated in the appropriate secondary antibodies at room temperature for 1 h. Subsequent to being washed, the blots were developed and gray scale scanning (iBox Scientia 500/600, UVP, Upland, CA, USA) was performed. The expression levels of LC3, PI3K, Akt, p-Akt, mTOR, and p-mTOR proteins were normalized to β-actin.

### Statistical analysis

All analyses were performed with SPSS 13.0 software (SPSS Inc., Chicago, IL, USA). The measurement data are presented as mean ± standard deviation (SD) and the multi-group comparisons were made with a one-way factor analysis of variance and the Least Significant Difference post hoc test. Values of P<0.05 were considered to indicate a statistically significant difference. The TEM image selection was performed randomly.

## Results

### Observation of autophagosomes under TEM

**Figure 1.**
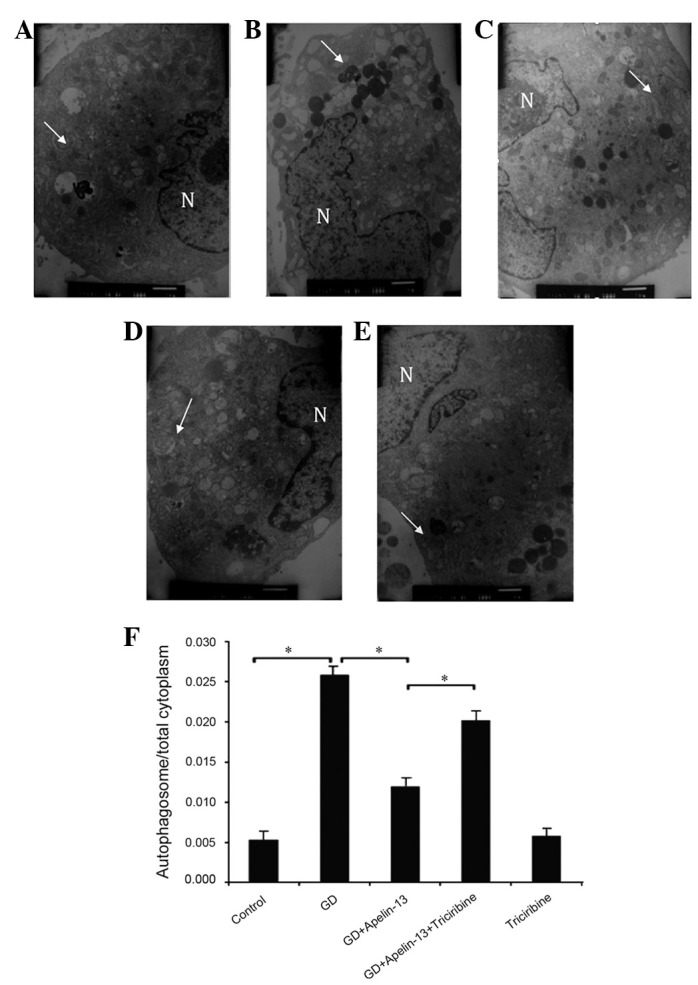
Myocardial cell autophagosomes under TEM (×8,000). (A) Co group, (B) GD group, (C) GD+Apelin-13 group, (D) GD+Apelin-13+Triciribine group and (E) Triciribine group; arrows indicate the bilayered membrane structure of the autophagosomes wrapped around the cytoplasmic components during cellular degradation. GD significantly increased the number of autophagosomes, while pretreatment with Apelin-13 significantly decreased the number of autophagosomes compared with the GD group. Using Apelin-13 and triciribine resulted in a significantly greater number of autophagosomes than when using Apelin-13 alone. There was no significant difference in autophagosome number between the Apelin-13 and Co groups. (F) Quantitation of the autophagosome to cytoplasmic ratio by group, which indirectly shows the degree of autophagy (^*^P<0.05). TEM, transmission electron microscopy; Co, control; GD, glucose deprivation; N, nuclei.

Autophagosomes are mono- or bilayer membrane structures that engulf regions of the cytoplasm that contain degraded or abandoned organelles. In the present study, the cardiomyocytes in each group contained varying numbers of autophagic vacuole-like structures under TEM. The number of autophagosomes in the GD group was significantly increased compared with in the Co group. The GD+Apelin-13 group had markedly fewer autophagosomes than the GD group. Conversely, the addition of triciribine, as in the GD+Apelin-13+Triciribine group, increased the number of autophagosomes compared with the GD+Apelin-13 group. There was no significant difference observed between the Triciribine and Co groups (P>0.05; Fig. 1).

### Autophagy-related protein LC3 expression

**Figure 2.**
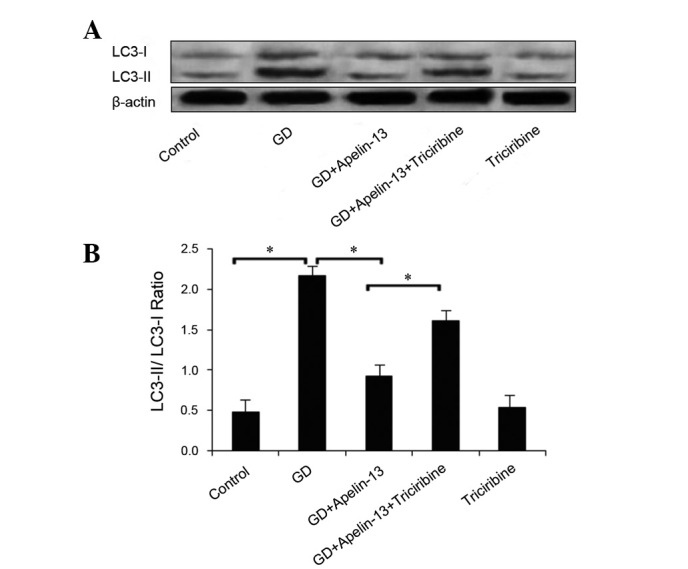
LC3 protein detection by western blotting. LC3 has two subtypes, LC3-I and LC3-II. When autophagy occurs, the conversion from LC3-I to LC3-II is significantly increased. (A) Immunoblotting of LC3-I and LC3-II. The conversion from LC3-I to LC3-II is apparent in the GD group, but was significantly decreased by the pretreatment with Apelin-13. The addition of triciribine improved the conversion of LC3-I to LC3-II. There was no significant difference in the conversion of LC3-I to LC3-II between the Triciribine and Co groups. (B) LC3-II band density normalized to LC3-I, showing the conversion rate of LC3-I to LC3-II. This indirectly shows the autophagy rate of the myocardiocytes. Data are expressed as mean ± standard deviation (SD), (n=6), ^*^P<0.05. LC3, light chain 3; GD, glucose deprivation; Co, control.

LC3 is an autophagic marker that exists in two forms: type I and type II. Prior to the occurrence of autophagy, LC3 is processed to form a soluble type I LC3 protein and autophagy induces further processing (ubiquitination) of LC3-I, inducing it to bind phosphatidyl ethanolamine on the surface of the autophagy layer to form type II LC3. The LC3-II/LC3-I ratios positively correlate with the autophagosome number (13), thereby providing an indirect measure of autophagy. The present study used the LC3-II/LC3-I ratio to assess the effect of Apelin-13 on LC3 protein expression; a larger ratio indicated enhanced autophagy in the mammalian cells. Fig. 2 shows the results of this analysis for each group. Compared with the Co group, the ratio of LC3-II/LC3-I in the GD group increased significantly (P<0.05). Conversely, the ratio was significantly decreased in the GD+Apelin-13 group compared with the GD and GD+Apelin-13+Triciribine groups (both P<0.05). A significant difference was not observed between the Triciribine and Co groups (P>0.05; Fig. 2).

### PI3K activity

**Figure 3.**
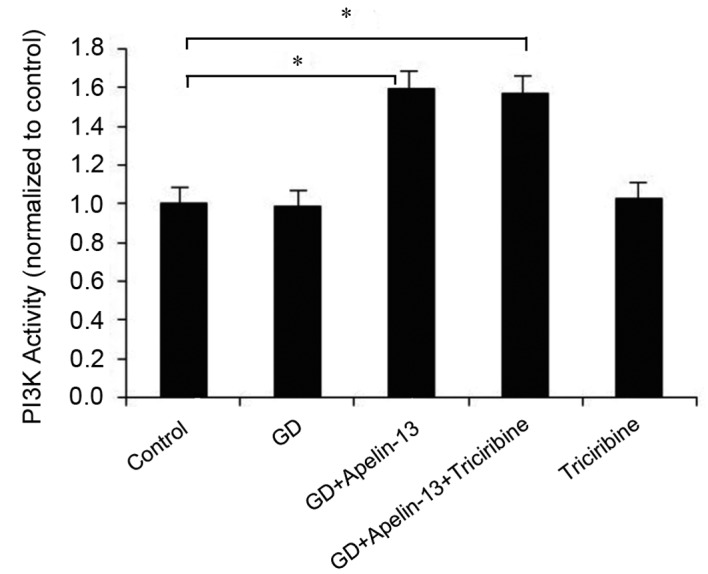
PI3K activity normalized to the control, showing that the PI3K activity was significantly increased by the pretreatment with Apelin-13 (^*^P<0.05). GD, glucose deprivation; PI3K, phosphatidylinositol-3-kinase.

PI3K activities in the GD+Apelin-13 and GD+Apelin-13 +Triciribine groups were significantly greater (1.59- and 1.56-fold greater, respectively) than those observed in the Co group. These differences were statistically significant (P<0.05). However, the PI3K activities in the GD and Triciribine groups were not significantly different from the Co group (P>0.05; Fig. 3).

### Expression levels of PI3K/Akt/mTOR pathway-related proteins

**Figure 4.**
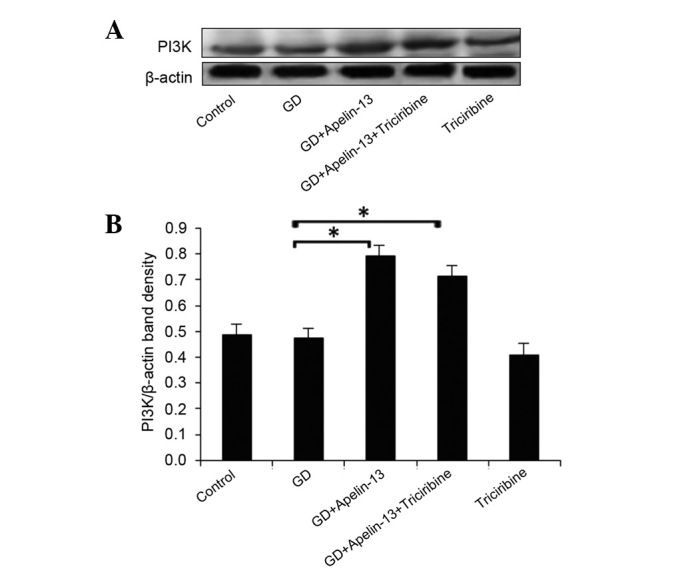
Detection of PI3K by western blotting. (A) Immunoblotting of PI3K, showing that the PI3K activity was significantly increased by the pretreatment with Apelin-13. (B) PI3K band density normalized to β-actin, ^*^P<0.05. GD, glucose deprivation; PI3K, phosphatidylinositol-3-kinase.

Compared with the GD group, PI3K and phosphorylated mTOR (p-mTOR) expression levels in the GD+Apelin-13 and GD+Apelin-13+Triciribine groups were significantly increased (P<0.05). There was no significant difference in PI3K expression between the GD+Apelin-13 and GD+Apelin-13+Triciribine groups (P>0.05), but p-mTOR expression was decreased significantly in the GD+Apelin-13+Triciribine group compared with the GD+Apelin-13 group (P<0.05; Fig. 4).

**Figure 5.**
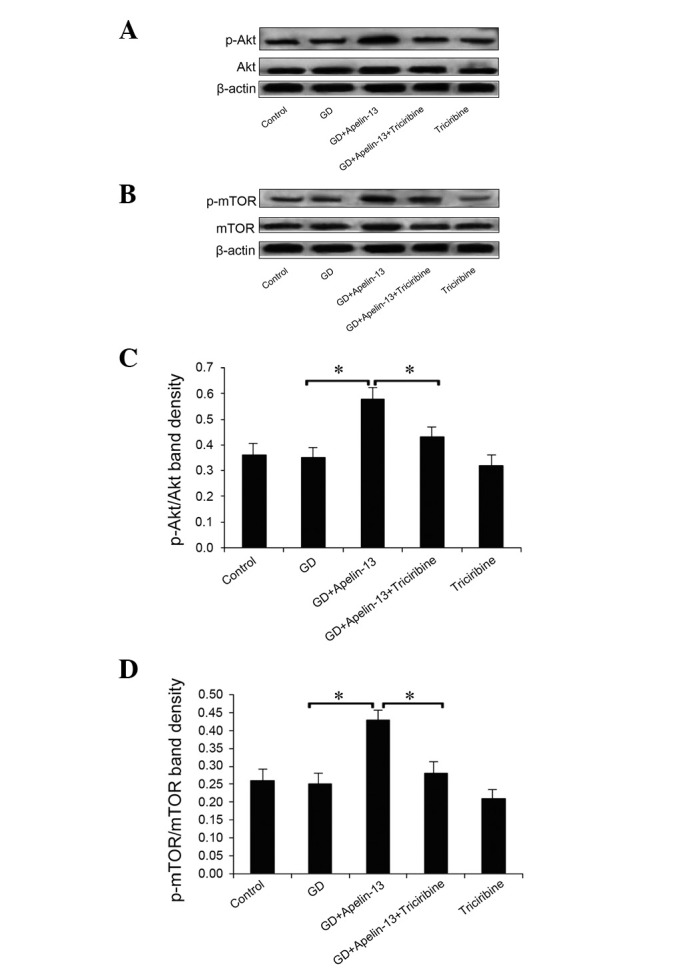
Akt, p-Akt, mTOR and p-mTOR protein detection by western blotting. (A) Immunoblotting of Akt and p-Akt. (B) Immunoblotting of mTOR and p-mTOR. No significant differences in the expression of Akt and mTOR were noticed among the groups. The pretreatment with Apelin-13 significantly increased the expression of p-Akt and p-mTOR, which was significantly decreased by the combined treatment of Apelin-13 and Triciribine. The graphical representations (normalized to unphosphorylated forms) of (C) p-Akt and (D) p-mTOR proteins. Data are expressed as mean ± standard deviation (n=6), ^*^P<0.05. mTOR, mammalian target of rampamycin; GD, glucose deprivation.

Compared with the Co group, the p-Akt and p-mTOR expression levels in the GD group were slightly, but not significantly, decreased (P>0.05). The Apelin-13 pretreatment significantly increased the p-Akt and p-mTOR levels (P<0.05), which were inhibited by the triciribine pretreatment (P<0.05). However, the total expression levels of the Akt and mTOR proteins were not significantly changed by the treatments (P>0.05; Fig. 5).

## Discussion

Autophagy occurs in eukaryotic cells at a low level under normal conditions. Cell autophagy may be initiated to remove damaged organelles when stimulated by ischemia and hypoxia, nutrient deficiency, oxidative stress, infection and other factors. Normally, autophagy is protective, but excessive activation of autophagy may lead to type II programmed cell death (2). Therefore, ensuring the timely and appropriate cessation of autophagy prevents injured cells from irreversible injury. In the present study, cardiomyocyte autophagy was induced by culturing the cell in a sugar- and serum-free culture medium (7).

Apelin was originally purified from bovine gastric secretions by Tatemoto *et al*(9) using a reversed pharmacological method. Apelin was ultimately determined to be the endogenous ligand of the orphan G protein-coupled receptor, APJ (14). Apelin is highly expressed in the cardiovascular system where it exerts protective effects, including the dilation of blood vessels to reduce blood pressure (15), improvements to heart function, the inhibition of myocardial ischemia injury (1) and the promotion of angiogenesis (16).

We previously demonstrated that Apelin-13 is able to inhibit GD-induced cardiomyocyte autophagy in a concentration-dependent fashion and that 1 *μ*mol/l Apelin-13 is the optimal concentration for this inhibition (7). Previously, it has been demonstrated that Apelin-13 is able to inhibit acute ischemia-induced myocardiocyte apoptosis by activating the PI3K/Akt singaling pathway (10). Other studies have shown that the small molecule triciribine is able to inhibit Akt activity (17,18). Therefore, in the present study cells were pretreated with 1 *μ*mol/l Apelin-13 and administered the Akt inhibitor triciribine 30 min prior to observing cardiomyocyte autophagy and protein expression levels to investigate whether Apelin-13 is able to inhibit excessive autophagy of the GD cardiomyocytes by altering PI3K-Akt signaling.

TEM imaging is the gold standard for assessing autophagy (19,20). In the cells with active autophagy, the present study observed damaged organelles with swelling or degenerated mitochondria, which were indicated by a vacuolar bilayer membrane-like structure around the mitochondria, as well as residual bodies in autophagic lysosomes that were not degraded. Calculating the ratio of autophagosomes to the total cytoplasmic area is considered as the appropriate method for quantifying autophagy. However, LC3 is the homolog of autophagy-related gene 8 (ATG8) and is therefore a useful indirect marker of autophagy.

It was reported that in cardiomyocytes cultured in GD medium, the ATP levels significantly decreased and the AMP/ATP ratios increased, which led to activation of AMP-activated protein kinase (AMPK). Moreover, activated AMPK inhibited mTOR in cardiomyocytes, which led to mTOR activation and to negatively regulated cell autophagy (1,13,21,22). In the present study, GD significantly increased the number of autophagosomes and the LC3-II/LC3-I ratio in neonatal rat myocardiocytes. Exogenous administration of Apelin-13 attenuated the GD-induced increase in the autophagosomes and the LC3-II/LC3-I ratio, indicating that Apelin-13 inhibited autophagy. Triciribine partially reversed the Apelin-13-mediated cardiomyocyte improvements, but triciribine alone did not exert any significant effect on cardiomyocytes compared with the Co group (P>0.05). Overall, Apelin-13 was observed to effectively inhibit cardiomyocyte autophagy and this effect was likely related to increased PI3K-Akt signaling.

The mechanism of autophagy is relatively complex and involves numerous diverse signaling pathways, including mTOR (4,23). mTOR is an evolutionarily highly conserved serine/threonine kinase which is involved in the regulation of cell responses in changed nutrition conditions and a number of energy-related regulatory pathways. mTOR is the gating mechanism of autophagy and negatively regulates the process (23). Therefore, mTOR activation may be indirectly inhibited using triciribine to inhibit Akt and promote autophagy. Activation of extracellular receptors leads to the phosphorylation of tyrosine kinase receptors and the phosphorylated sites recognize and combine with the p85 subunit of PI3K to activate type I PI3K signaling. This in turn produces phosphatidylinositol 4,5-bisphosphate and 3,4,5-trisphosphate to activate Akt, which activates mTOR and inhibits autophagy (24). In the present study, the PI3K activity and the levels of p-Akt and p-mTOR were significantly increased by Apelin-13, however, the effect was partially inhibited by triciribine. Combined with the TEM results, these results lead to the hypothesis that Apelin-13 inhibited cardiomyocyte autophagy by activating the PI3K-Akt signaling pathway and by simultaneously activating the downstream signaling molecule mTOR.

Autophagy plays a significant role in immunity, infection, inflammation, tumors and cardiovascular and neurodegenerative diseases (25). There are numerous studies with regard to the involvement of autophagy in tumors, neurodegeneration and immune conditions, but it is relatively less studied in cardiovascular diseases. In the present study, the mechanisms underlying the regulatory effect of Apelin-13 on GD-induced autophagy in cardiomyocytes were assessed. The results confirmed that Apelin-13 is able to partially attenuate GD-induced autophagy by increasing the expression levels and activation of the components of the PI3K/Akt/mTOR signaling pathway. These findings suggest that Apelin-13-related drugs may provide new methods for protecting cardiomyoctyes from injury, but full understanding of the specific pathways that inhibit autophagy requires further study.
